# Projecting the real through active inference: a unified model of reality construction from neuronal dynamics to social consensus *a neuroscientific framework for psychopathology*

**DOI:** 10.3389/fpsyt.2026.1815025

**Published:** 2026-10-14

**Authors:** Panov G., Panova P.

**Affiliations:** 1 Psychiatric Clinic, University Hospital for Active Treatment “Prof. Dr. Stoyan Kirkovich”, Trakia University, Stara Zagora, Bulgaria; 2 Department of Neurology, Psychiatry and Psychology, Government University “Prof d-r Asen Zlatarov”, Burgas, Bulgaria; 3 Medical Faculty, Trakia University, Stara Zagora, Bulgaria

**Keywords:** active inference, default mode network, precision-weighting, predictive coding, psychopathology, reality construction, reality projection, social synchronization

## Abstract

How does the brain transform a chaotic stream of sensory signals into a stable, shared experience of reality? This theoretical paper proposes that the brain does not passively receive the world, but actively projects it – it continuously generates predictions about sensory inputs and updates its internal model when predictions fail, a process formally described by the free energy principle and active inference. We argue that this process can be understood through an analogy with quantum decoherence – the collapse of multiple possibilities into a single actuality. By integrating insights from this analogy with active inference, we show how the brain resolves sensory uncertainty into a coherent projection of reality. A central question we address is whether projecting reality changes it. We argue that it does: a strong, coherent projection reduces uncertainty, while a weak or fragmented one amplifies prediction errors, and a distorted one – diverging from social consensus – leads to alternative realities that resist correction. This projection operates in discrete, rhythmic cycles governed by brain waves and is fine-tuned by neurochemicals that determine how much we “trust” our predictions versus new sensory data. The Default Mode Network – the brain’s inner “sea” of self-awareness, memories, and context – acts as a central hub that integrates and updates these projections. Crucially, individual projections are stabilized into a shared, “solid” reality through social interaction, language, and culture – a process we call collective active inference. Within this framework, psychiatric disorders are understood as disorders of projection – distortions in how the brain generates and stabilizes its model of reality. This perspective reverses the traditional explanatory gradient: psychopathology is not merely a sensory or chemical problem, but a fundamental disruption of the brain’s capacity to project a coherent world. This unifying framework offers a new foundation for both pharmacological and psychotherapeutic interventions, grounding them in a common computational logic.

## Introduction: from sensory noise to projected reality

How does the brain turn a chaotic stream of sensory signals into a stable, coherent experience of the world? This question has puzzled philosophers and scientists for centuries. In recent decades, a powerful answer has emerged: the brain is not a passive receiver of information, but an active hypothesis-generator. It continuously generates predictions about what is about to happen, compares them with incoming sensory evidence, and updates its internal model when predictions fail (1–3). This is the core idea of predictive coding and active inference – a framework that has proven heuristically fruitful in computational psychiatry (4, 5).

In this paper, we build on this framework to propose a unifying concept: reality projection. We argue that what we call “perception” is not a direct reflection of the world, but a top-down construction – a projection – that the brain generates and continuously refines. This projection is not passive; it is actively constructed, shaped by prior beliefs, and stabilized through social interaction. When this projection becomes distorted – whether by aberrant weighting, fixation on traumatic priors, or chaotic network dynamics – psychopathology emerges.

At every moment, the brain is bombarded by a sensory stream of billions of bits of information. Estimates suggest that while the entire sensory apparatus receives on the order of 11 million bits per second (6) only a tiny fraction – roughly 16 to 50 bits per second – reaches conscious awareness, a cognitive bottleneck that reflects the structured bandwidth limitations of the conscious workspace (7). This reduction of information represents an active process of filtration: the brain filters out over 99.999% of incoming information, amounting to a reduction by more than a factor of 100,000 (8, 9). What determines the degree of filtration and the permeability of the filter? Two principal groups of factors: (1) genetic predisposition, which sets the baseline tone of filtration (10, 11), and (2) epigenetic changes induced by accumulated experience – trauma, enriched environments, or chronic stress – which permanently alter gene expression, fine-tuning the filter’s permeability and shaping dominant predictive models (3, 12).

We argue that the reduction of sensory uncertainty and the minimization of prediction errors are two sides of the same phenomenon: the continuous construction of a stable perceptual reality. This reality is associated with a synthesis of a neurocognitive substrate, analogous to the reconstruction of memory traces; while the immediate perceptual alignment occurs via rapid electrochemical computations, the long-term stabilization and storage of these constructed models into enduring traces structurally depends on *de novo* protein synthesis (13–15).

## Extending the framework: the common logic of resolution

The reduction of sensory uncertainty – the process by which the brain selects one interpretation from many possible ones – and the minimization of prediction errors – the correction of mismatches between predictions and actual sensory input – are not merely parallel processes. They reflect a common logic for solving an information overload problem by imposing order.

One way to grasp this logic is through an analogy with quantum physics. In quantum mechanics, a particle exists in a superposition of many possible states until it interacts with its environment – a process known as decoherence – which “collapses” the possibilities into a single actual state (16, 17). We propose that the brain performs a functionally similar operation: it continuously faces a range of possible interpretations of sensory input, and through hierarchical predictive coding, it resolves this uncertainty into a single, coherent percept – a projection. This analogy is not meant to imply that the brain is a quantum computer, but to illustrate a general principle: reality, whether physical or perceptual, emerges from the reduction of possibilities to actualities. This insight echoes a rich tradition of proposals suggesting that quantum principles and cognitive architecture share deep formal relationships, spanning from abstract structural similarities to radical quantum-biological models of mind (18, 19). This raises a deeper question: if reality is a projection, do we not then perceive what we have already projected? The observer effect in quantum mechanics suggests that observation itself participates in the constitution of the observed state. If the brain operates analogously, then perception and projection may not be sequential but mutually constitutive – a circularity that lies at the heart of our model.

In neuroscience, this maps precisely onto active inference: the brain minimizes variational free energy – an information-theoretic measure of surprise – by updating its generative model to better predict sensory inputs (2, 4). This can be understood as a form of belief updating, where the brain continuously revises its predictions based on new sensory evidence (20). This leads to a further question: could the minimization of variational free energy be understood as an analogue of quantum decoherence – a process by which the brain selects one interpretation among many, thereby constituting its current reality? We do not claim this as a literal equivalence, but as a heuristic parallel that may illuminate the logic of active inference.

The physical environment and the biological sensory stream serve as similar sources of uncertainty – both must be reduced through Bayesian inference to yield stable descriptions of reality. What we add is the explicit proposal that the resolution of uncertainty is the computational analogue of confidence-weighted prediction error minimisation and that both are expressions of a more general principle: the active construction of reality through the reduction of uncertainty.

Crucially, this common logic operates at multiple levels – spanning from molecular mechanisms to neural circuits, and from individual perception to social synchronization and is modulated by the same neurochemical systems that encode confidence and prediction errors. The remainder of this article explores these levels in turn, moving from theoretical principles to clinical applications.

## Discrete cycles: the temporal architecture of reconstruction

Active filtration does not occur continuously but within discrete, cyclically repeating windows synchronized with brain rhythms such as alpha (8–12 Hz) and theta (4–7 Hz) (21, 22). The alpha rhythm, for instance, functions as a periodic attentional filtering mechanism (23). Each phase represents a “sampling window” for sensory input, followed by a window for internal processing – a window filled with predicted information (24). Within the active inference framework, these rhythmic cycles correspond to discrete updates of the generative model: periods of sensory sampling followed by belief updating and policy selection – the process of choosing actions based on current predictions (25).

This discrete logic provides a neurophysiological substrate for the analogy: brain filtration reduces chaos into rhythmically organized packets that are “assembled” into a smooth perceptual experience. The gaps between them are not empty but are filled with predictions and internal models generated by the Default Mode Network (DMN) (2). This rhythmic organization of perception and cognition echoes Jean Piaget’s classical concepts of assimilation and accommodation—principles which modern psychological frameworks, such as Therapeutic Assessment, directly utilize to describe how the mind adapts its internal predictive structures to new experiences while maintaining coherence (26). Disruptions to this rhythmic organization – e.g., anomalies in alpha synchronization – can lead to a breakdown in the fluidity of reconstruction (27). It is no coincidence that distinct alterations in alpha frequency are observed across psychiatric disorders: schizophrenia is characterized by reduced power and desynchronization, correlating with symptoms of disorganization (28); epilepsy manifests in paroxysmal, uncontrolled bursts of synchronization (29); and depression has been associated with altered resting frontal alpha asymmetry, although the direction and magnitude of this association have shown substantial variability across studies (30). Our own work has further demonstrated that alpha frequency dysrhythmia is associated with treatment-resistant schizophrenia, suggesting that persistent instability of this rhythmic filtering mechanism may underlie the failure to stabilize a coherent reality in severe psychosis (31).

A complementary framework to rhythmic sampling is provided by the discrete two-stage model of perception (32). According to this view, a long period of non-conscious processing (hundreds of milliseconds) precedes discrete conscious perception. During this unconscious window, the brain integrates, segregates, groups, and weights sensory elements. At the end of this window, information from temporary memory is “collapsed” into a conscious percept, and the internal generative model is updated. Crucially, no conscious experience occurs without such updating. The absence of perceived gaps between discrete conscious moments is explained by the fact that the intervening processing is unconscious.

This framework aligns closely with our active inference model: the unconscious window corresponds to the period during which prediction errors are accumulated and hierarchical belief updating occurs, while conscious perception marks the moment of model revision. The varied spatiotemporal resolution of these windows may also support the philosophical view that consciousness itself is fundamentally discrete – a form of controlled “hallucination” updated at discrete time points.

## Molecular foundations: memory as an active reconstructive process

The rhythmic structure of perception is not merely an electrophysiological phenomenon; it is grounded in the same molecular machinery that underpins memory and learning. Just as discrete waves organize sensory input, discrete molecular events – such as protein synthesis and synaptic consolidation – organize the storage and retrieval of the projections we call memories.

Crucially, the way the brain filters and resolves sensory uncertainty is not neutral – it is shaped by prior experience stored in memory. The brain does not resolve the world from a blank slate; it does so from a position of accumulated priors, which determine which interpretations are privileged and which are suppressed. This view is consistent with the cognitive-perceptual theory of memory, which holds that early memories function as cognitive blueprints that may shape present perception and expectation (33). Perception, in this sense, is always a memory-informed projection.

Perceptual reconstruction is deeply interwoven with the dynamics of memory. The emotionally charged memories utilized by the DMN are themselves products of a reconstructive process. Each act of recall involves the re-activation of neural circuits and *de novo* protein synthesis for restabilizing (reconsolidation) or modifying the memory trace (34, 35). The process of perceptual synthesis and its subsequent encoding can be described as a parallel to the mnemonic one: sensory information from occipital, temporal, and other analysers converges in the hippocampal-entorhinal complex, where, within the present context, it is synthesized into a coherent scene – a process whose long-term storage is dependent on protein synthesis. In exactly the same way, during recall, fragments of past events are recombined in the same complex, but this time under the influence of current perception and emotional tone. The result is always a new, situationally altered “mix” of past fragments and present context, which again requires protein synthesis for reconsolidation (14, 36).

This parallel between perceptual synthesis and mnemonic reconstruction supports the philosophical and neurological idea of the “perennial now.” Just as perception is not an instantaneous snapshot but a synthesis of information from milliseconds in the past into a coherent model (the theory of the “momentary present” (37, 38):), so too is memory always a reconstruction performed within the context of the present moment (39). Both processes are projective acts that create a sense of continuity, despite being based on discrete neural processing. In PTSD, this process may be disrupted, fixing the traumatic memory in a maladaptive, structurally rigid form that resists standard updating and contaminates current perceptual reconstructions (40–42).

This perspective raises a further possibility: remembering is itself a form of projection. When we recall an event, we see it, hear it, and feel as though we are inside the memory. The brain synthesizes new proteins and we “enter” the recollection – a top-down projection that reconstructs the past in the present. In this view, we are constantly shifting between different projections: the ongoing perceptual projection of the here-and-now, and the mnestic projection of the remembered there-and-then. The overlap and switching between these projection modes may be central to understanding dissociative phenomena and certain features of post-traumatic stress disorder, where the traumatic memory intrudes as an alternative, competing projection.

## Active inference and generative models

How does the brain decide which projections to generate and which to suppress? And how does it use these projections to guide action? This is where active inference provides the computational scaffold – a formal framework for understanding how the brain selects policies and actions to minimize surprise, thereby shaping the very reality it projects.

Active inference provides the formal foundation for our model. It extends predictive coding by treating action as a way of sampling the environment to test and fulfill predictions (25, 43). Under this framework, the brain not only infers states of the world but also selects policies (action sequences) that minimize expected free energy – balancing epistemic value (information gain) and pragmatic value (goal attainment).

Recent work on hierarchical generative models has shown that such architectures can successfully support continual learning through generative replay (44), flexible cognitive intentions (45) and the scalable, complex motor control of bodily movements (46). These mechanisms are essential for maintaining stable yet adaptive projections across time.

Top-down predictions shape even low-level perceptual processes such as binocular depth estimation, illustrating the pervasive influence of the generative model across the sensory hierarchy (47). Within the active inference framework, the brain continuously generates predictions about sensory inputs – a process we identify with “projection”. These predictions are tested against actual sensory data, and the resulting prediction errors drive model updating. Critically, this predictive dynamics is part of a continuous loop of reality construction. From this perspective, reality is not discovered but actively projected, with each cycle of prediction and revision refining the next projection.

## The role of the DMN: the turbulent sea of context

The policies and predictions generated by active inference are not computed in a vacuum. They are shaped by the brain’s most powerful integrative structure – the Default Mode Network – which embodies our highest-level priors about self, world, and others. This network serves as the central hub where sensory streams, memories, and emotional valences converge to generate a coherent projection of reality.

To understand this integration, we can envision the Default Mode Network (DMN) as a turbulent, moving “inner sea” – the background of self-awareness, memories, and mental simulations (48, 49). The stream of filtered sensory information (the “river”) flows into it and is immediately dispersed, mixed, and reinterpreted by its currents – the dominant internal context (emotion, thought). In active inference terms, the DMN embodies our highest-level priors – the most abstract and stable beliefs about ourselves and our world (50). These deep temporal models generate predictions that cascade down the cortical hierarchy, shaping perception and action. The DMN is the chief integrator, assigning meaning to the incoming information.

Pathology arises when this “sea” becomes too turbulent and overwhelming (as in OCD), too stagnant (depression), or detached from the incoming “river” (derealization). The DMN’s ability to maintain a stable context depends on its dynamic interaction with the Salience Network (SN), which determines the weight of stimuli that pass through – i.e., which prediction errors are trusted and thus drive belief updating – and switches activity between the DMN and the Executive Network (EN) for goal-directed action (51, 52). When this balance is disrupted, the DMN either dominates excessively (as in depression, where negative priors overwhelm new evidence) or fails to provide a stable context (as in schizophrenia, where the “inner sea” becomes chaotic) (51). This connection between DMN dynamics and specific symptom profiles provides a direct link between our computational model and clinical phenomena.

Building on this, the DMN can be viewed as a central hub where sensory streams are continuously compared with ongoing predictions, and where the resulting percept is integrated with emotional valence and self-relevance. In this view, the DMN does not simply reflect past experiences but actively participates in the projection of reality, updating its priors with each new cycle of sensory input. The emotional valence assigned to stimuli (via the salience network) determines which aspects of the projection are prioritized for action and which are suppressed. Thus, the DMN operates as a dynamic filter that balances bottom-up sensory evidence with top-down projections, constantly re-weighting the contribution of each according to the current context and emotional state.

## The neurochemical orchestra: mediators of confidence, prediction error, and reconstruction

But a network, however sophisticated, is only as good as the modulatory signals that tune it. The confidence in predictions and prediction errors is not fixed; it is dynamically regulated by a neurochemical orchestra – glutamate, acetylcholine, dopamine, and serotonin – each encoding a different aspect of confidence, surprise, and salience. We now turn to this chemical fine-tuning of the projection process.

The construction and maintenance of perceptual reality are orchestrated by the dynamic interplay of key neuromodulator systems, which in active inference encode the confidence or weight assigned to predictions and prediction errors (11, 43).

Within the active inference framework, it is hypothesized that glutamate conveys prediction errors up the cortical hierarchy (53–55). Consistent with this view, ketamine – an NMDA receptor antagonist – disrupts glutamatergic transmission, which has been interpreted as altering the weighting of sensory evidence and effectively decoupling bottom-up signals from top-down predictions (54–56). This provides a pharmacological manipulation of the generative model’s belief updating, though alternative interpretations of ketamine’s effects exist.

According to active inference, acetylcholine is thought to modulate the reliability of sensory information, determining which prediction errors are trusted and thus drive learning (57, 58). The major packets of cholinergic neurons innervating the cortex are located in the basal forebrain (nucleus basalis of Meynert) and the brainstem (pedunculopontine nucleus) (59).

It has been proposed that dopamine encodes confidence-weighted prediction errors related to reward expectations and uncertainty, thereby signalling the need to update policies (60, 61). Antipsychotics (D2 receptor antagonists) are hypothesized to reduce the weight of aberrant salience, thereby helping to re-stabilize consensual reconstruction (62, 63). This interpretation aligns with the aberrant salience theory of psychosis, though direct evidence linking dopamine dynamics to confidence-weighting in humans remains indirect.

It has also been proposed that serotonin modulates the global tone of the system, balancing exploration and exploitation by adjusting the weight of prior beliefs about the environment (64). Selective serotonin reuptake inhibitors (SSRIs) may gradually refine this weighting, thereby altering negative contextual biases in depression. However, in some cases, this has been associated with emotional blunting – potentially reflecting excessive confidence in emotional nuance (65, 66). These interpretations remain theoretical and require direct experimental testing.

From the perspective of active inference, therefore, pharmacological interventions might be understood as potential modulators of confidence at different levels of the cortical hierarchy (67, 68). This framing generates testable hypotheses. For example, one could design a study where participants perform a sensory attenuation task before and after administration of a dopamine antagonist, measuring changes in confidence-weighted prediction errors via EEG or fMRI. Alternatively, a clinical trial could compare the effects of SSRIs and placebo on confidence-weighting parameters in depressed patients, using behavioral tasks that assess belief updating in response to positive and negative feedback.

Thus, manipulating specific neurotransmitter systems may affect not only predictive coding (i.e., the projection of expectations) but also the weighting of sensory streams and their integration within the DMN. From a clinical perspective, this suggests that pharmacological interventions (e.g., antipsychotics, SSRIs, ketamine) could be understood as modulators of the balance between top-down projection and bottom-up sensory processing, rather than simply correcting a “chemical imbalance”. This dual action – on both prediction and sensory weighting – may explain why drugs with similar receptor profiles can have divergent clinical effects depending on the patient’s prior beliefs and current context. However, this remains a hypothesis to be tested empirically.

## Boundary cases: oneiroid states and dreams as windows into the mechanism

The neurochemical orchestration of confidence is not merely a theoretical construct; its breakdown is most visible in extreme states where sensory input is severely attenuated or internally overridden. These boundary conditions – oneiroid states and dreams – reveal what happens when the balance between projection and sensory input collapses, and will serve as a testing ground for our framework.

The strength of the model is evident in boundary conditions. In the oneiroid syndrome, sensory input is severely attenuated, while the internal activity of the DMN and limbic circuits becomes hyper-dominant. In active inference terms, this represents a collapse of the weighting balance: sensory confidence approaches zero, while prior confidence dominates completely. Reality is reconstructed almost entirely from internal predictions, stabilizing into an alternative, pathologically coherent state (69). Dreams offer a parallel: they demonstrate that even without external input, consciousness operates using already filtered and consolidated material from memory – with dream imagery, including human faces, being reconstructed from elements of past visual experience (70) – supporting the thesis of working with archived material under reduced prefrontal control (71, 72). This does not mean that dreams are identical to oneiroid states or that they represent a “scheme” of psychopathology; rather, they illustrate the same underlying principle of internal model dominance in the absence of strong sensory input.

This analysis suggests a direct parallel between the oneiroid state and the pharmacological action of ketamine, which induces oneiroid-like experiences by reducing sensory flow through NMDA receptor blockade. More broadly, between normal waking consciousness and the full oneiroid state there exists a continuum of intermediate conditions characterized by graded reduction of sensory confidence and graded increase of DMN/salience network dominance. In oneiroid states proper, the patient actively projects a reality, acts within it, and responds to its internally generated demands – they live inside their projection. Recognizing this continuum may help bridge phenomenological and neurocomputational accounts of psychosis.

## Normality as a dynamic equilibrium

The oneiroid state and dreams reveal the extremes of projection – where internal models dominate over sensory input. But normal waking consciousness lies between these extremes, a dynamic equilibrium between receptivity and projection, between the external world and the internal sea of context. We now examine this equilibrium and its disturbances in psychopathology.

Normal waking consciousness represents an optimal, dynamic equilibrium between hyper-receptivity to external signals (fragmentation) and hyper-dominance of the internal model (oneiroid states). From an evolutionary perspective, this system evolved to balance two primary modes of allostatic regulation: (1) an environmentally-driven Scanning/Searching mode (where sensory precision is elevated to detect threats or resources) and (2) an internally-driven Focusing/Execution mode (where stable prior predictions suppress unneeded sensory noise to pursue specific behavioral goals) (73, 74). For instance, metabolic demands like hunger fundamentally alter predictive weighting, forcing the brain to project a highly motivated, resource-centered model of the world, which dynamically shifts toward a rest and consolidation mode (DMN dominance) once homeostasis is restored. This dynamic equilibrium relies on the continuous adjustment of internal models through assimilation and accommodation—shifting weights to fit new experiences while maintaining overall cognitive coherence (26).

This dynamic equilibrium, or “getting stuck” in a mode, finds its neural correlate in the concept of neural flexibility. The healthy brain demonstrates a high capacity for rapidly switching between different functional network states in response to internal or external demands (75, 76). We can view psychopathology as a sustained deviation from this dynamic equilibrium – a “stuckness” in one of the modes, manifesting as reduced network flexibility or entrenchment. For example, depression is characterized by hyperconnectivity and dominance of the DMN, hindering the switch to task-related networks (77), while schizophrenia exhibits excessive dynamics and chaotic switching, reflecting a lack of stable projection (78).

## Symbolic naming as a socio-cognitive act of stabilization

But equilibrium is not only a neural affair. It is also shaped by the symbolic systems we use to name, categorize, and share our projections. Language – the most powerful of these systems – transforms private experience into shared reality, and in doing so, stabilizes or destabilizes the projections we live by. We now turn to this socio-cognitive act of stabilization.

The fundamental mechanism for crystallizing experience into a shareable reality is symbolic naming (primarily through language). Within the active inference framework, this act corresponds to the acquisition of discrete categorical priors that shape perception (79, 80). Naming transforms a continuum of impressions into a discrete, thinkable category – effectively creating a new prior in the generative model. This act is not passive; it performs a back-projection onto perception itself. Naming something as “danger” changes its weighting and how you perceive it. This finds support in empirical evidence for linguistic influence (or modern linguistic relativism), where language categories shape perception and categorization (81, 82). This process resonates with self-verification theory, which proposes that individuals actively seek out and interpret experiences that confirm their existing self-concept (83).

This process is also informed by the philosophy of Ludwig Wittgenstein, who argued that “the limits of my language mean the limits of my world” (84). At the collective level, this process of shared naming creates “linguistically constructed realities,” which become the foundation for social synchronization (85). Socialization is the internalization of these collective priors (86), which synchronize individual generative models into a common semantic framework.

This raises a deeper question: is language itself the primary medium through which uncertainty is reduced at the social level? If the reduction of uncertainty is the collapse of multiple possible interpretations into a single actualized meaning, then naming performs precisely this operation on the level of meaning. Before a phenomenon is named, it exists in a state of interpretive ambiguity – it could be many things. The act of naming resolves this ambiguity into a single, socially sanctioned category. However, the meaning of a name is not fixed; it varies across individuals and contexts. For example, the word “love” may mean very different things to different people, yet it still serves as a shared reference point. In this sense, language is not merely a tool for describing a pre-existing reality; it is a mechanism for constituting reality by selecting one interpretation among many and stabilizing it through shared use, while still allowing for variation in personal meaning. This linguistic stabilization is the foundation of culture, norms, and collective projection – and its failure may underlie certain forms of psychopathology, particularly those involving delusional or idiosyncratic meaning-making.

## The projective nature of reality: reconstruction as external projection

Once named and shared, projections gain a social reality that transcends the individual mind. They become part of a collective framework – a consensual reality that we inhabit together. But this externalized world is not a mere passive reflection; it is an active projection, continuously constructed and reconstructed by the brain. We now turn to this projective act itself.

A radical but logical conclusion follows: we do not merely perceive reality; we project it. The brain is an active hypothesis-generator that constructs the most probable model (6, 87). Input data are meaningless until they are resolved and projected back into a coherent spatial representation as an objective world. This projection is internal neural activity that bestows qualities.

Consider colour perception. We do not see colours as they “really are” in the physical world – we see wavelengths of light that our brain translates into colours. The same physical wavelength is perceived as green, blue, or red depending on the context, the state of the visual system, and even the language we speak. In this sense, colour is not a property of the external world but a projection of the brain – a linguistic-cognitive construction that transforms a continuous physical spectrum into discrete, named categories. This projection is so seamless that we mistake it for direct perception, yet it is a prime example of how the brain resolves a continuum into a stable, meaningful reality. The same principle applies to sounds, tastes, and even social meanings – we project order onto noise, and that order becomes our world.

This view is not merely philosophical; it is empirically grounded. Neuroimaging studies have shown that higher visual areas (V4 and VO1) represent the colour we see rather than the physical wavelength of light entering the eye, indicating a transition from a physical stimulus to a perceptual construct (88, 89). In a classic demonstration of top-down projection, viewing a grayscale image of a banana activates the neural representation of yellow in primary visual cortex (V1), suggesting that prior knowledge about the world is projected back onto early sensory areas (90). These findings align with the active inference framework: perception is an inside-out process of projection, not an outside-in registration of stimuli (5, 91).

The projective character of reality is evident not only in perception but also in how our expectations shape social and experimental reality. This is illustrated by the experimenter effect (92). In a famous experiment, researchers who believed they had “maze-bright” and “maze-dull” rats (actually randomly assigned) reported significantly better performance for the “bright” rats due to unconscious differential treatment (92). This is analogous to the reverse Pygmalion effect (Golem effect) in a social context, where low expectations lead to poorer outcomes (93), and the original Pygmalion effect, where high expectations improve them (94). These phenomena demonstrate that projection is not merely an internal mental event: it manifests in behaviour and shapes the physical and social environment, creating a confirmation cycle. This raises a fundamental question: do we perceive and then update our predictions about the world, or do we project first and then perceive what we have projected? Or are both processes simultaneously at work, raising the question of their temporal sequence? We suggest that projection and perception are mutually constitutive – two sides of a single, ongoing process of reality construction. This circularity is precisely what makes reality so difficult to disentangle from the process of projecting it. Therefore, ‘reality’ is a projected reconstruction – a continuous act of resolution, naming, and social synchronization.

## Social decoherence as collective active inference: synchronizing projections into a “Solid” reality

If individual projection is the foundation of subjective reality, its stabilization into a shared, consensual world requires a collective act. We now turn to this process – social decoherence – where individual projections synchronize through language, ritual, and shared attention, creating the solid ground we call “culture” and “common sense”.

This projected reality becomes stable and “solid” through the process of social (intersubjective) decoherence, which we reframe as collective active inference. Through shared language, common rituals—which have been shown to induce profound physiological synchronization among participants (95)—and behavioral mimicry, individual generative models are aligned to minimize shared uncertainty.

As Hohwy (4) articulates, self-organizing systems that possess Markov blankets – boundaries separating internal and external states – will appear to infer and act on their world to preserve integrity. At the collective level, groups form higher-order Markov blankets, synchronizing their beliefs through shared narratives. This creates a consensual, shared reality – an analogue of a stable state on a group scale, representing a vital mechanism for brain-to-brain coupling (96). Culture and socialization thus operate as a durable history of collectively constructed realities, transforming the cultural origins of human cognition (97) into a shared semantic framework (98). This collaborative construction is essential for stability; when this shared predictive alignment fails, the world can become overwhelming or systemically distorted (99).

Crucially, the consensus reality that emerges from collective active inference is not universal. Different cultures construct different shared realities, with varying norms regarding attention, emotion regulation, self-concept, and social perception. This cultural variability has given rise to transcultural psychiatry – a discipline that emphasizes the role of cultural context in shaping what is considered normal or pathological. What is perceived as a “distorted projection” in one cultural setting may be a perfectly adaptive or even celebrated projection in another. This does not imply that all projections are equally valid, but rather that the boundary between healthy and disordered projection is partly determined by the cultural consensus within which the individual is embedded. Recognizing this variability is essential for avoiding the over-pathologization of culturally normative differences and for developing culturally sensitive diagnostic and therapeutic approaches.

The process of collective projection is not always successful. When it fails, it can lead to profound social isolation, a phenomenon that has profound clinical and epidemiological implications for modern well-being (100). Autism Spectrum Disorder (ASD) offers a compelling illustration of this breakdown: it can be understood as a fundamental difference in the weighting of social signals, which disrupts the synchronization of individual projections with the social group. Cognitively, individuals with ASD often show a high and inflexible precision assigned to sensory details, which reduces their ability to extract global social context (101). Neurally, this predictive misalignment is closely linked to alterations in the mirror-neuron system (102) and decreased inter-brain synchrony during live face-to-face interactions (103). As a result, their projections remain less automatically aligned with the consensual social construction – manifesting as difficulties in joint attention, theory of mind, and language. In this sense, psychopathology can emerge either as a failure to synchronize, mirroring how Bayesian imbalances construct false beliefs in conditions like schizophrenia (104), or as the forcible imposition of a single projection, as observed in totalitarian dynamics”.

## An integrative psychopathological model: breakdowns in the multilayered process

Social synchronization of projections is not always successful. When it fails, it not only creates isolation but can also lead to enduring distortions of projection that shape psychopathology. We now turn to how our framework unifies these disturbances into a coherent model of mental disorders.

If reality is a projection, then psychopathology is, at its core, a disorder of projection – a breakdown in the brain’s ability to generate, stabilize, and synchronize a coherent projection of the world. Within this framework, two distinct types of projection failure can be identified. The first is a weak or fragmented projection – one that fails to provide a stable, coherent model of the world, leading to high prediction errors and perceptual instability. The second is a distorted projection – one that is stable and coherent, but diverges from the socially constructed consensus reality. This type of projection does not generate high prediction errors internally, but rather a persistent mismatch with the shared realities of others. Both types can lead to psychiatric disorders, but they manifest differently and may require different therapeutic approaches.

It is important to recognize that these distortions are not simply “errors” to be corrected. They often represent the individual’s best attempt to adapt to overwhelming circumstances – a protective strategy that prioritizes survival or meaning-making over accuracy. This perspective does not deny the suffering associated with these conditions, but it reframes psychopathology as a human response to adversity, rather than a mere malfunction of the brain.

Schizophrenia represents a profound disruption in the predictive hierarchy, where the mis-calibration of confidence between priors and sensory evidence breaks down the boundary separating internal projections from external reality (105). Sterzer et al. (105) propose that psychosis dynamics involve an unstable shifting of precision, where aberrant salience (62) forces the system to generate hyper-dominant, idiosyncratic internal models to compensate for chaotic prediction errors (106). The result is a systemic failure where alternative realities stabilize as the patient’s only coherent defense (107). Corollary discharge mechanisms further specify this account: inappropriate efference copy signals flood the sensory systems, disrupting the predictability of one’s own actions and making the system prone to delusions of control and illusions of external agency (108).

Importantly, schizophrenia illustrates both types of projection failure. The weak or fragmented projection manifests primarily as negative symptoms – apathy, avolition, emotional blunting, and social withdrawal. These are not merely “absent” functions, but reflect a failure of the brain to generate a stable, coherent projection of the world, leading to pervasive uncertainty and high prediction errors. In contrast, distorted projection manifests as positive symptoms – delusions and hallucinations. Delusions, whether persecutory, erotomanic, jealous, or grandiose, represent stable but socially non-consensual projections. The patient projects a reality that is internally coherent but diverges from the shared reality of others. This distinction is clinically relevant: negative symptoms are notoriously resistant to antipsychotic treatment, whereas positive symptoms often respond to dopamine antagonists, which reduce the weight of aberrant salience (62, 63). In our framework, antipsychotics may thus be understood as modulators that reduce the confidence in distorted projections, while the treatment of weak projections may require different strategies – perhaps those that strengthen the generative model itself, such as cognitive remediation or psychosocial interventions.

Depression is a projection dominated by negative priors. Pathologically confident negative expectations dominate inference, making positive prediction errors insufficient to update beliefs (109). The DMN’s chronic negative context tints all new perceptions, creating a self-fulfilling cycle of hopelessness. Here, the projection is not weak but stable – it is distorted toward negativity, diverging from the consensual reality that others perceive.

This distorted projection is not a random cognitive error, but a well-documented attentional negativity bias: the depressive brain selectively filters out positive and neutral information while amplifying negative stimuli (110). Clinically, this means that the patient does not merely “feel” sad, but literally projects a world from which all positive cues have been excised – the smile on a friend’s face is missed, a neutral comment is interpreted as a personal attack, and the future is seen as devoid of hope. Crucially, because this bias operates as a core maintaining mechanism of the disorder, clinical interventions must actively disrupt these rigid internal projections; empirical evidence demonstrates that structured, collaborative frameworks—such as Therapeutic Assessment—can effectively challenge and reconfigure these deeply entrenched negative models within clinical settings (111).

Crucially, this biased projection is often an adaptive response to overwhelming life circumstances, which shifts the brain’s computational mechanisms toward a pattern of biased belief updating (112). For many individuals, these negative priors accurately reflect experiences of loss, failure, or social exclusion. The depressive projection may therefore serve as a protective strategy – a way of anticipating and preparing for further pain. In this context, therapeutic interventions, such as cognitive-behavioural therapy (CBT), function as collaborative efforts to re-weight these priors and broaden the range of possible interpretations. This process closely aligns with the principles of Therapeutic Assessment (111) which emphasize the collaborative exploration of meaning rather than the simple “correction” of cognitive distortions”.

PTSD is a projection stuck in the past. Traumatic priors are fixated with abnormally high confidence, preventing reconsolidation and imposing traumatic structure on present inference (113). The world is seen through the lens of past trauma, transforming the somatic and cognitive reality of the individual as the body chronically keeps the score of past danger (114).

This fixation is not merely a failure of memory; it is a protective mechanism. By keeping the traumatic priors hyper-precise, the brain attempts to avoid future danger – a form of active inference that prioritises survival over accuracy. However, this comes at a cost: the traumatic projection overwrites present experience, making it difficult to perceive safety or change. Therapeutic approaches such as EMDR or exposure therapy can be understood as interventions that reduce the confidence in these traumatic priors, allowing the projection to be updated and reconsolidated in the light of new, non-threatening experiences. This process is inherently collaborative and requires a safe therapeutic relationship, a clinical reality that is foundational to the efficacy of Therapeutic Assessment (111).

Anxiety is a projection oriented toward future threat. Over-confident predictions about future dangers drive excessive policy selection for avoidance behaviors, where the brain errs on the side of caution by sacrificing accuracy for safety (115). This hyper-vigilant projection narrows the individual’s world, excluding non-threat possibilities through a mis-calibrated insular processing of anticipatory uncertainty (116).

Therapeutic interventions, such as cognitive-behavioural therapy (CBT) or mindfulness-based approaches, function as robust tools to recalibrate these anxious projections. Rather than simply dismissing the existence of external threats, these modalities expand the individual’s repertoire of responses and systematically increase the confidence assigned to benign predictions. Within collaborative paradigms like Therapeutic Assessment, this process is operationalized not as a simple correction of errors, but as a shared, relational reconfiguration of the patient’s rigid future-oriented models.

Autism Spectrum Disorder is a projection that fails to synchronize with social signals. Atypical confidence in social priors and sensory signals (100, 101) leads to a filter that is too permeable for sensory details but too rigid for global social context, hindering joint attention, theory of mind, and language.

Intentionality complements this framework: disruptions in the directedness of mental states toward objects or goals – a failure of intentionality – can be understood as collapses in expected free energy over goal-directed policies (117).

From this viewpoint, therapies are interventions in the projection process: re-weighting confidence through pharmacological means, modulating priors, and reconsolidating traumatic memories via eye-movement and exposure protocols (118, 119). Ultimately, re-synchronizing the individual’s generative models with the collective requires collaborative social and psychological interventions that rebuild shared meaning, a process exemplified by systemic frameworks like Therapeutic Assessment (111).

## Limitations: normative assumptions, adaptive confidence, and cultural variability

The framework we have outlined is powerful but not without boundaries. In this section, we address its limitations – the normative assumptions it carries, the adaptive value of confidence shifts, and the cultural variability that shapes what we call “disorder”.

The framework we have presented assumes that deviations from a “balanced” confidence-weighting profile constitute psychopathology. However, this assumption requires careful qualification for at least three reasons.

First, adaptive confidence shifts. In some contexts, what appears as a confidence imbalance from a statistical norm may actually be adaptive. For example, individuals with a history of trauma may develop heightened threat sensitivity (i.e., overly confident threat priors) that reduces the likelihood of re-exposure to danger – a form of active inference that successfully shapes behavior and environment. Similarly, in environments characterized by objective adversity or exploitation, “negative priors” may accurately track real conditions rather than reflecting a cognitive distortion. As critics of purely pathological accounts have noted, what looks like a deviation from a “balanced” brain state may sometimes represent a rational adaptation to adverse circumstances (9, 10). Our model does not equate statistical deviation with dysfunction; rather, we follow standard psychiatric definitions that require distress, impairment, or risk of harm. The same computational profile may be adaptive in one context and maladaptive in another.

Second, cultural variability. As we have discussed, the consensus reality that emerges from collective active inference is not universal – different cultures construct different shared realities, with varying norms regarding attention, emotion regulation, self-concept, and social perception. While our framework acknowledges this variability, it does not yet provide a formal mechanism for predicting which cultural priors will lead to adaptive versus maladaptive projections. In other words, we can describe that culture shapes projection, but we cannot yet specify how or when a given cultural consensus will produce a “distorted” projection relative to another. This is a critical direction for future research. Cross-cultural studies in computational psychiatry are still nascent, and we caution against overgeneralizing findings from culturally homogeneous baseline templates, as human attention and genetic baselines inevitably constrain how different populations process information (9, 10). The development of culturally sensitive diagnostic and therapeutic approaches will require not only empirical data but also a refined computational model that can incorporate cultural priors as modulators of confidence and belief updating.

This perspective is consistent with recent developments in Therapeutic Assessment, a collaborative approach that views psychological symptoms not merely as deficits to be corrected, but as meaningful adaptations that can be explored and understood within a relational and systemic context (111, 120). Such approaches remind us that even when a projection is “distorted” relative to social consensus, it may represent the patient’s best attempt to make sense of an overwhelming experience. From a practical standpoint, this raises the question of how clinicians can assess these processes in their daily work. One promising avenue is the use of performance-based personality tests, such as the Rorschach, interpreted through the lens of active inference. Meyer and Friston (121) have argued that the Rorschach task provides a unique window into the Bayesian brain’s meaning-making processes, offering a way to observe how individuals resolve sensory ambiguity and project their internal models onto unstructured stimuli. A poignant clinical example is provided by Kpassagou (122), who describes a case in which psychotic symptoms served as a protective solution against intolerable representations, illustrating how a “distorted” projection can be understood as an adaptive – if costly – strategy”.

Third, environmental causation vs. internal dysfunction. A computational description of aberrant confidence does not, by itself, identify the cause of that aberration. Environmental factors – chronic stress, poverty, exploitation, trauma – may induce confidence changes that are rational responses to adverse conditions. In such cases, the primary locus of intervention may be environmental rather than neuropharmacological or psychotherapeutic. Our framework is compatible with this view: it describes mechanisms, not ultimate causes, and does not claim that all confidence deviations reflect “broken brains”.

We have incorporated these considerations into the model not as afterthoughts but as essential boundaries on its scope and claims. Future work should test whether the confidence parameters we discuss vary systematically across cultures and contexts, and whether adaptive confidence shifts can be distinguished from truly maladaptive ones using behavioral and neural markers.

## Conclusion and future directions

With these limitations in mind, we now bring together the threads of our argument – from the reduction of sensory uncertainty to social synchronization – and outline the implications of the projective model for epistemology, clinical practice, and future research.

The present work offers a synthesizing model that bridges the traditional divides between physics, neuroscience, psychology, and sociology in explaining the fundamental phenomenon of reality construction. Through the integration of the quantum analogy with active inference – the neurocomputational framework of predictive coding and confidence-weighting – we demonstrate that stable, everyday experience is not a given but a dynamic, active act of projection. The brain is not a passive receiver but a hypothesis-generating system that continuously minimizes prediction errors to construct a coherent narrative, which is then externalized and stabilized within the group through collective active inference.

As Goethe intuited, “A man sees only what he knows” – but we would add: a man projects what he knows, and through shared projections, we build a common world.

A central question arises from this framework: if reality is a projection, does the act of projecting it change it? And if so, does a weak or fragmented projection lead to higher prediction errors – and ultimately to psychopathology? We suggest that the answer is yes. A strong, coherent projection reduces prediction errors by actively shaping expectations and filtering sensory input. Conversely, when the projection is weak – whether due to aberrant confidence-weighting, traumatic fixation, or chaotic network dynamics – the brain is flooded with unresolved prediction errors. This is not a failure of perception; it is a failure of projection. Psychopathology, in this view, is not a distortion of an objective reality, but a disruption of the very process by which reality is constructed. The therapeutic implication is profound: treatment should aim not only to correct sensory misperceptions, but to strengthen the patient’s capacity to project a stable, coherent world.

The primary implications of this model are threefold:

1. Epistemological (Theory of Knowledge): It challenges naïve realism, proposing that “reality” is always mediated by biological and social filters – i.e., by the confidence structure of our generative models. This is not solipsism but projective realism grounded in active inference, where shared reality is a product of collective belief updating.
2. Clinical (Psychopathology): It offers a unifying paradigm that translates the disparate symptoms of mental disorders into a common denominator: disruptions in confidence-weighting within hierarchical active inference. This directly guides treatment toward correcting the specific breakdown in the hierarchy – from molecular modulation of confidence to social synchronization of generative models.
3. Methodological (Future Research): The model generates testable hypotheses that integrate these hierarchical breakdowns with the genetic predispositions and developmental gene-environment interactions that anchor the computational psychiatry framework (11, 12) For example: Can measures of neural synchronization (e.g., alpha coherence) predict treatment response by indexing confidence-weighting parameters? Do therapies targeting memory reconsolidation (e.g., EMDR) lead to measurable changes in the confidence of traumatic priors as inferred from behavioral tasks? Can the “strength” of social synchrony in a given culture – conceptualized as the degree of shared priors – correlate with the population prevalence of specific mental disorders? Does deep kinematic inference expand this computational architecture, providing a vital bridge for understanding how motor projections actively shape perceptual reality in health and disease (8). We acknowledge that these predictions are derived from the framework rather than already empirically confirmed. Direct tests would require (i) operationalizing confidence parameters in behavioral or EEG tasks (e.g., through sensory attenuation paradigms or hierarchical Bayesian modeling of response times), (ii) demonstrating that pharmacological manipulations (e.g., ketamine, SSRIs, antipsychotics) alter these parameters in predicted directions, and (iii) showing that individual differences in confidence-weighting predict clinical outcomes and treatment response. Such tests remain a priority for future research”.

Most psychopathological conditions are traditionally understood as disorders of perception or reality distortion, placing emphasis on bottom-up (sensory) mechanisms – a “down-top” gradient of explanation. In the present framework, we reverse this gradient. We propose that many psychiatric phenomena are better understood as primary disruptions of projection – i.e., of top-down expectation and prior weighting – rather than as simple sensory misrepresentations. From this viewpoint, psychopathology is not merely a failure to perceive reality correctly but a failure to project a reality that remains aligned with consensual norms and adaptive goals. This shift from a bottom-up to a top-down explanatory gradient has direct implications for treatment: interventions (pharmacological, psychotherapeutic, or social) may be most effective when they target the confidence and content of projections, rather than attempting to “correct” sensory input alone.

The model of “projected reality through active inference” does not merely describe how the mind works – it offers a new cartographic projection for the scientific territory. It invites neuroscientists to consider quantum analogies, computational psychiatrists to examine neurochemical orchestrations of confidence, and sociologists to investigate collective belief updating through the lens of active inference. In this interdisciplinary perspective lies the promise not only of a deeper understanding of mental health but also of a more complete answer to the question of how an organism, adrift in a sea of noise and probabilities, manages to build and share a stable, meaningful world through the relentless minimization of surprise.

A final, fundamental question remains: do we not perceive what we have already projected? If we draw on quantum mechanics, observation itself – always situated within our knowledge and experience – participates in constituting what is observed. Perception and projection may thus form a virtuous circle, each chasing the other. It is this very circularity – the continuous interplay of projecting and perceiving – that we may call time and life.

## Data Availability

The original contributions presented in the study are included in the article/supplementary material. Further inquiries can be directed to the corresponding author.
